# A hybrid AI model integrating BKA-VMD and deep neural networks for industrial power load prediction

**DOI:** 10.1371/journal.pone.0329630

**Published:** 2025-08-14

**Authors:** Yin Luo, Chaofan Guo, Minfeng Pan, Hong Zhou

**Affiliations:** Electronics and Information College, Liuzhou Polytechnic University, Liuzhou, China; Air Force Engineering University, CHINA

## Abstract

Accurate power load prediction is crucial for optimizing energy consumption and enhancing efficiency in industrial environments. However, the highly nonlinear and non-stationary nature of power load time series presents significant challenges. To address this, we propose a novel hybrid deep learning model that integrates optimized data decomposition with advanced sequence modeling to enhance feature extraction and temporal pattern learning. Specifically, Variational Mode Decomposition (VMD) optimized by the Black-Winged Kite Algorithm (BKA) extracts intrinsic mode functions, reducing noise and improving signal representation. The decomposed signals are processed by a hybrid neural network combining a One-Dimensional Convolutional Neural Network (1DCNN) for local feature extraction, a Bidirectional Temporal Convolutional Network (BiTCN) for long-range temporal dependencies, a Bidirectional Gated Recurrent Unit (BiGRU) for sequential pattern learning, and an attention mechanism to emphasize critical features. Extensive experiments, including comparisons with state-of-the-art models and ablation studies, validate our approach across three diverse industrial datasets. The results demonstrate that our model significantly outperforms existing methods, achieving lower Mean Absolute Error (MAE) and Root Mean Square Error (RMSE). The ablation study highlights the critical roles of the attention mechanism and the BiTCN-BiGRU combination in capturing complex temporal dependencies. These findings underscore the model’s robustness and adaptability for power load forecasting. Future research should focus on enhancing generalization and validating applicability across diverse industrial settings.

## 1. Introduction

Recently, the rising costs of raw materials and energy consumption during processing have prompted increasing attention from industrial enterprises towards cost reduction and efficiency improvement. These enterprises aim to optimize the allocation of processing energy and enhance resource utilization through effective strategies. In the machinery industry, where the production process involves the fabrication of mechanical components, the energy consumption of production line equipment is significant. A crucial approach for improving efficiency while maintaining production output is to strategically plan energy usage and organize production schedules to minimize unnecessary electricity waste.

Power load forecasting is pivotal in production planning within the industrial sector. Numerous studies have investigated forecasting techniques specifically tailored to industrial power demand. For instance, Liu et al. [1] introduced a hybrid approach that integrates TimeGAN, CNN, and LSTM, enhancing the accuracy of short-term load predictions for both industrial and commercial buildings. Similarly, Zhu et al. [2] applied a day-ahead forecasting strategy, leveraging load change rate features and combining the firefly algorithm with machine learning models to predict industrial load demands. Furthermore, research [3–6] has highlighted the effectiveness of integrating machine learning algorithms with data preprocessing techniques, such as signal processing, to improve forecasting precision in industrial contexts. These developments underscore a growing trend toward hybrid methodologies in power load forecasting, which aim to provide more robust and reliable predictions for industrial applications.

Industrial electricity demand is characterized by seasonal variations that can significantly influence load forecasting accuracy. Time series decomposition has emerged as a key technique to address these fluctuations. Widely adopted methods for decomposition include Empirical Mode Decomposition (EMD) [7], VMD [8], and Complete Ensemble Empirical Mode Decomposition with Adaptive Noise (CEEMDAN) [9]. For example, Zheng et al. [10] employed an EMD-LSTM model for short-term load fore-casting, while Lv et al. [11] utilized a VMD-LSTM hybrid model to forecast power grid loads. Similarly, Luo et al. [12] introduced a forecasting method that combined CEEMDAN with TCN-LSTM. Wang et al. [13] integrated STL and multi-head self-attention in deep learning model for multi-step runoff forecasting. New developments have been achieved in the field of enhancing decomposition methods for more accurate predictions. Aswanuwath et al. [14] proposed determining the appropriate decomposition level of EMD to guide the VMD process, thereby eliminating the need for filtering analysis and reducing both computational time and cost. Furthermore, Wang et al. [15] optimized VMD parameters using the bald eagle search algorithm, which improved the quality of time series decomposition. Huang et al. [16] introduced a parameter optimization strategy lever-aging the sparrow search algorithm, which, when integrated with feature selection techniques, significantly boosted the accuracy of short-term load forecasting. Fan et al. [17] proposed a hybrid model that combines GWO optimized VMD algorithm with CNN-BiLSTM model, to make more accurate prediction on power load when the power system is highly complex. However, smart grids face increasing security threats, such as false data injection attacks (FDIA), therefore Tiann et al. [18,19] demonstrating their proposed models impact on state estimation and deep learning detection systems.

Optimizing parameters to improve decomposition outcomes has become a critical challenge, prompting the integration of optimization algorithms with decomposition methods. In recent decades, there has been a growing focus on leveraging computational models to understand key processes in nature, such as survival, adaptation, and competition, alongside the progression of natural phenomena. This focus has been extended to both the natural and social sciences, causing the progress in a number of optimization techniques. Prominent algorithms include the Gravitational Search Algorithm (GSA) introduced by Rashedi et al. in 2009 [20], as well as socially-inspired methods like the Teaching-Learning-Based Optimization (TLBO) algorithm suggested by Rao et al. in 2011 [21]. These advances were guided by the “No Free Lunch Theorem” (NFL) [22], which asserts that no optimization method excels in all scenarios, high-lighting the need for context-specific approaches. This principle has catalyzed the creation of more specialized algorithms, with several new optimization methods emerging in recent years [23–32]. These techniques have significantly enhanced the accuracy of forecasting models, with particular attention given to the Black-winged Kite Algorithm(BKA), introduced by Wang et al. in 2024 [33], which has shown superior performance compared to previous methods.

Machine learning approaches have gained significant popularity in load forecasting research. Traditional forecasting methods, such as ARIMA [34–35], Support Vector Regression (SVR) [33], and Multi-Layer Perceptron (MLP) [36,37], have long been utilized. However, given the non-linear nature of time series data, deep learning models have become increasingly favored in recent studies. Models like Convolutional Neural Networks (CNN) [38,39], Long Short-Term Memory (LSTM) networks [40,41], and Transformers [42–44] have been widely adopted for power load forecasting. To capture local features within time series data, researchers have also integrated multiple models with attention mechanisms to boost forecasting accuracy. For example, Liu et al. [45] proposed a hybrid model that cope with CNN, bidirectional LSTM (BiLSTM), and an attention layer, which significantly enhanced forecasting performance. Luo et al. [46] introduced a stacking ensemble algorithm in accordance with CNN-BiLSTM-Attention and XGBoost, applied to a multi-feature load dataset from the Quanzhou area (2016–2018). Their comparative analysis shown a cut in the Mean Absolute Percentage Error (MAPE) by 5.88–69.40%. Song et al. [47] incorporated spatiotemporal attention with a gated temporal convolutional network to enable multi-task learning, reducing MAPE by up to 25.09%, 25.96%, and 17.46% for cooling, heating, and electrical load forecasting tasks, respectively. Fan et al. [48], building on the DTformer model, proposed the Temporal Top Windowed Attention (TWA) module and the Dual Variable Attention module to address extended temporal and inter-variable dependencies, yielding promising forecasting results on a campus power load dataset. Wang et al. [49] proposed a model which combined the singular spectrum analysis(SSA) with the attention-based BiTCN model, and the method can be used for trend and periodicity analysis of runoff data, providing an important basis for hydrological management.

This paper presents a novel hybrid deep learning model for power load forecasting in industrial environments, addressing the challenges posed by the nonlinear and non-stationary nature of power load time series. The main contributions of this work are as follows:

1. Optimized data decomposition: We introduce a Variational Mode Decomposition (VMD) method optimized by the Black-Winged Kite Algorithm (BKA) to decompose the time series into intrinsic mode functions (IMFs), enhancing signal representation and reducing noise.
2. Advanced hybrid neural network architecture: Our model integrates 1D Convolutional Neural Networks (1DCNN) for local feature extraction, Bidirectional Temporal Convolutional Networks (BiTCN) for capturing long-range temporal dependencies, and Bidirectional Gated Recurrent Units (BiGRU) for sequential pattern learning, all enhanced by an attention mechanism to focus on critical features.
3. Empirical validation: Through extensive experiments, including comparisons with state-of-the-art models and ablation studies, we demonstrate that our approach achieves superior prediction accuracy, with significant reductions in Mean Absolute Error (MAE) and Root Mean Square Error (RMSE). The proposed model not only advances the state of the art in power load forecasting but also provides a robust framework that can be adapted to other time series forecasting tasks in smart grid applications Table 1.

**Table 1:** Abbreviation list.

Abbreviation	Full Name
1DCNN	One-Dimensional Convolutional Neural Network
AM	Attention Mechanism
ARIMA	AutoRegressive Integrated Moving Average
BKA	Black-Winged Kite Algorithm
BiGRU	Bidirectional Gated Recurrent Unit
BiLSTM	Bidirectional Long Short-Term Memory
BiTCN	Bidirectional Temporal Convolutional Network
CEEMDAN	Complete Ensemble Empirical Mode Decomposition with Adaptive Noise
CNN	Convolutional Neural Networks
EMD	Empirical Mode Decomposition
FDIA	False Data Injection Attacks
GSA	Gravitational Search Algorithm
GRU	Gated Recurrent Unit
IMFs	Intrinsic Mode Functions
LSTM	Long Short-Term Memory
MAE	Mean Absolute Error
MAPE	Mean Absolute Percentage Error
MLP	Multi-Layer Perceptron
NFL	No Free Lunch Theorem
RMSE	Root Mean Square Error
RNN	Recurrent Neural Network
SSA	Singular Spectrum Analysis
SVR	Support Vector Regression
TCN	Temporal Convolutional Network
TLBO	Teaching-Learning-Based Optimization
TWA	Temporal Top Windowed Attention
VMD	Variational Mode Decomposition
GWO	Grey Wolf Optimizer
FA	Firefly Algorithm
ELM	Extreme Learning Machine
KOA	Kepler Optimization Algorithm
WOA	Whale Optimization Algorithm
ALO	Ant Lion Optimization
GWO	Grey Wolf Optimizer
DA	Dragonfly Algorithm
MFO	Moth-Flame Optimization

## 2. Related methodologies

### 2.1. Black-winged kite algorithm(BKA)

The Black-winged kite algorithm(BKA) [33] is an optimization technique that uses meta-heuristics and draws inspiration from the predatory and migrating habits of the black-winged kite. It aims to emulates the black-winged kites’ intelligent foraging strategies and interactions which are dynamic and social, making it particularly effective for addressing complex optimization problems. In BKA, potential solutions are represented as individual “kites” within the search space. The algorithm progresses iteratively, simulating the kites’ movement as they search for food. This process is characterized by two primary stages: exploration and exploitation. Aiming at attaining a balance between global search capabilities and local refinement, both stages are quite critical in ensuring effective convergence to optimal solutions.

The algorithm mainly mimics the black-winged kite’s attack and migration behaviors. The iterative process of the algorithm can be outlined in the following manners:

By generating a couple of random solutions at first, a black-winged kite within the search space can be denoted within such solutions. This ensures that the initial population is diverse by spreading these positions evenly across the search space. At the same time, the optimal kite position is represented by the solution with the best fitness value, which is chosen as the leader (denoted as $X_{L}$). The random initialization is carried out using the following function:


$$Y_{i}={BKA}_{lb}+rand({BKA}_{ub}-{BKA}_{lb})$$ (1)

Here, ${BKA}_{lb}\;$and ${BKA}_{lb}$ represent the lower and upper bounds of the *i*-th black-winged kite in the *j*-th dimension respectively, while rand is a random value selected from the interval [0,1].

The initial leader of the kites’ group can be confirmed by solving the minimum value via the following formulas:


$$f_{best}=\text{min}(f(Y_{i}))$$ (2)

$$X_{L}=X(find(f_{best}==f(X_{i})))$$ (3)

#### 2.1.1. Attack behavior.

**Fig 1:**
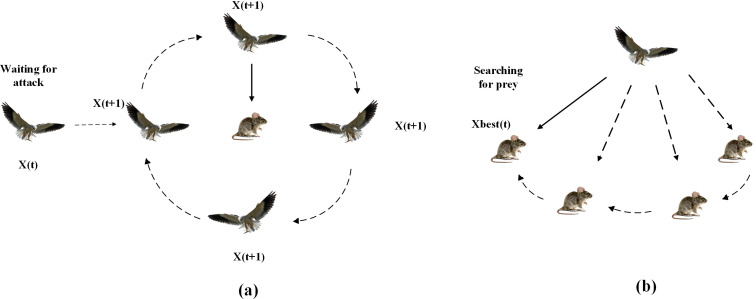
Conceptual diagram of two attack strategies of Black-winged kites (a) waiting for attack and (b) searching for prey.


This behavior models the predation process of the black-winged kite. Fig 1a presented that initially the kite scan for preying by hovering in the air, once the prey is located, it will dive down and attack the prey fleetly. This behavior is abstracted into two forms of actions: global exploration and local search. By employing sine and exponential functions in the mathematical model, this attack strategy enables the algorithm to rapidly converge towards potentially optimal regions within the search space. The following is the mathematical model of the attacker’s activity:

$$y_{t+1}^{i,j}=\left\{ \begin{array}{c} y_{t}^{i,j}+n\left(1+\sin(r)\right)\times y_{t}^{i,j}\;p<r\\ y_{t}^{i,j}+n\times (2r-1)\times y_{t}^{i,j}\;else \end{array}\right.\;$$ (4)

$$n=0.05\times e^{-2\times {\left(\frac{t}{T}\right)}^{2}}$$ (5)

The variables $y_{t}^{i,j}$ and $y_{t+1}^{i,j}$represent the position of the *i*-th black-winged kite in the *j*-th dimension at the *t*-th and (t + 1)-th iteration steps, respectively. *r* refers to a random number that ranges between 0 and 1, and *p* refers to a constant value set to 0.9. *T* denotes the total number of iterations, and *t* refers to the number of iterations that have been completed up to the current step.

#### 2.1.2. Migration behavior.

**Fig 2:**
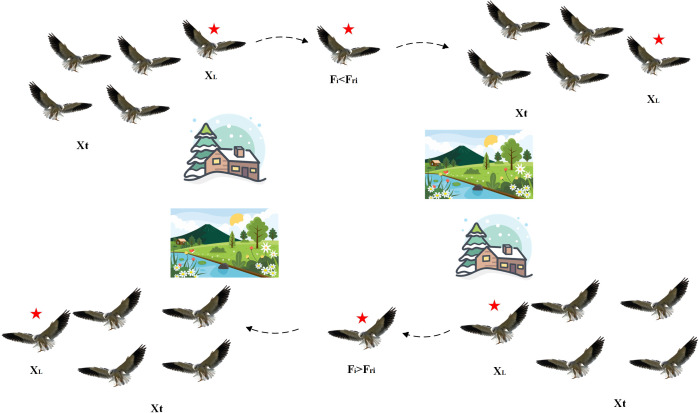
A conceptual diagram illustrating the aggressive and migratory behaviors of the black-winged kite.


This behavior mimics the black-winged kite’s migratory strategy when facing seasonal changes. As illustrated in Fig 2, when environmental conditions shift or food becomes scarce, the kite engages in mass migration. When the present population’s fitness value is below a random population’s, the leader gives up its position and mi-grates with the rest of the group. Alternatively, the leader continues guiding the population if its fitness value remains superior. This dynamic selection of the leader ensures successful migration, helping to escape local optima and guiding the search towards the global optimum. The mathematical formulation for migration behavior is as follows:

$$x_{t+1}^{i,j}=\left\{ \;\;\;\begin{array}{c} x_{t}^{i,j}+C(0,1)\times {(x}_{t}^{i,j}-L_{t}^{j})\;F_{i}<F_{ri}\;\\ x_{t}^{i,j}+C(0,1)\times \left(L_{j}^{t}-n\times x_{t}^{i,j}\right)\;else \end{array}\right.\;$$ (6)

$$n=2\times \text{sin}(r+\frac{\pi }{2})$$ (7)

### 2.2. Variational Mode Decomposition(VMD)

Variational Mode Decomposition(VMD) is a versatile data composition technique designed to decompose complex signals into multiple Intrinsic Mode Functions (IMFs), each corresponding to distinct frequency bands that capture the signal’s local oscillatory characteristics. Unlike the traditional Empirical Mode Decomposition (EMD), VMD offers improved numerical stability and superior resistance to noise. When the signal contains a lot of non-stationary or nonlinear components, VMD is particularly effective in dealing with such components, providing more reliable and accurate decompositions [8].


$$\begin{array}{c} \left\{ \begin{array}{c} {min}_{\left\{u_{k}\right\},\{\omega_{k}\}}\left\{\sum {\mathrm{\backslash nolimits}}_{k=1}^{k}{\left\|\partial_{t}[\left(\partial (t)+\frac{j}{\pi t}\right)*u_{k}(t)]e^{-j\omega_{k}t}\right\|}_{2}^{2}\right\}\;\;\;\;\;\;\;\;\;\;\;\;\;\;\;\;\;\;\;\;\;\;\;\;\;\;\;\;\;\;\;\;\;\;\;\;\;\;\;\;\;\;\;\;\;\;\\ s.t.\sum {\mathrm{\backslash nolimits}}_{k=1}^{k}u_{k}=f\; \end{array}\right.\; \end{array}$$ (8)

In this equation, $u_{k}$ denotes the segmented modal component of the Intrinsic Mode Function (IMF), andω represents the central frequency corresponding to the *k*-th component, δ(t) is the unit shock function. The symbol * indicates the convolution operation.

### 2.3. Bidirectional time convolution network(BiTCN)

A TCN represents a specific class of convolutional neural networks that leverage dilated convolutions to enlarge the receptive field, in which enabled effective modeling of long-range dependencies within time-series data. The operation for dilated convolution is as follows:


F(t)=(W⨀G)*X(t)\] (9)

This convolution kernel dictates the sampling interval from the input sequence, while the dilation factor adjusts the kernel’s sampling rate, thus controlling the receptive field’s extent. In TCNs, the length of the output from the convolutional layer generally matches the input, which allows these networks to efficiently process sequences of varying lengths.

**Fig 3:**
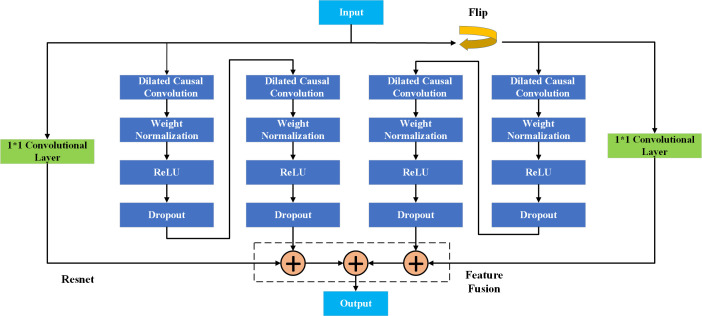
Bidirectional time convolution network architecture.

Incorporating a bidirectional processing mechanism, the BiTCN network extends the capabilities of the TCN by facilitating the extraction of features from time-series data in both forward and reverse temporal directions. As illustrated in Fig 3, the input sequences pass through two distinct TCNs: one processes the sequence in a forward direction, and the other in reverse. The results from both directions are then combined to produce the final output. Additionally, to address challenges such as gradient vanishing and explosion, residual concatenation is employed.

The forward TCN performs convolution from the beginning to the end of the sequence, generating the forward feature sequence. In contrast, the backward TCN conducts convolution in the reverse order, producing the backward feature sequence. Given an input sequence $X\in R^{T\times D}$, where 𝑇 represents the length of the sequence and 𝐷 stands for the feature dimension, the outputs of both TCNs can be represented as:


$$\vec{F}={TCN}_{forward}(X)$$ (10)

$$\overset{\leftarrow }{F}={TCN}_{backward}(X)$$ (11)

Where $\vec{F}\in R_{T\times C}$ and $\overset{\leftarrow }{F}\in R_{T\times C}$ denote two different direction of TCN output feature sequences, including a forward one and a backward one respectively, and C denote the feature dimension of the output.

Finally, the outputs from both the forward and backward networks are merged to yield the final result, which can be expressed as:


$$F=[\vec{F},\overset{\leftarrow }{F}]\in R^{T\times 2C}$$ (12)

### 2.4. 1D Convolutional Neural Network(1D-CNN)

One-dimensional Convolutional Neural Networks (1D-CNNs) are specifically designed for processing sequential data, for example, time-series or text data, where the primary structure is aligned along a single axis. In 1D-CNNs, the convolutional kernel (or filter) operates by sliding along this one-dimensional input to identify patterns within the sequence. If the data is represented as a vector $\left[x_{1},x_{2},\ldots x_{n}\right]$, the kernel traverses this vector to detect local features across the sequence. The kernel itself is a 1D array with a defined size k, which determines the number of elements the kernel spans at each step. In terms of time-series applications, the kernel moves along the time axis, capturing temporal dependencies within the data. The receptive field of a 1D-CNN kernel corresponds to a contiguous segment of the input sequence. As the kernel slides across the sequence, it aggregates information from k, consecutive input elements, enabling the network to learn local patterns in the data. The convolution operation in a 1D-CNN layer can be expressed as [47]:


$$(x*w)(t)=\sum_{i=0}^{k-1}x(t+i)\cdot w(i)$$ (13)

Where x represents the input sequence, w is the convolutional kernel, (x*w)(t) denotes the convolution of x and w at the position t, k refers to the size of the convolutional kernel, x(t+i) denotes the element of the input sequence at position t+i, and w(i) refers to the element of the kernel at position i.

### 2.5. Bidirectional gated recurrent unit(BiGRU)

**Fig 4:**
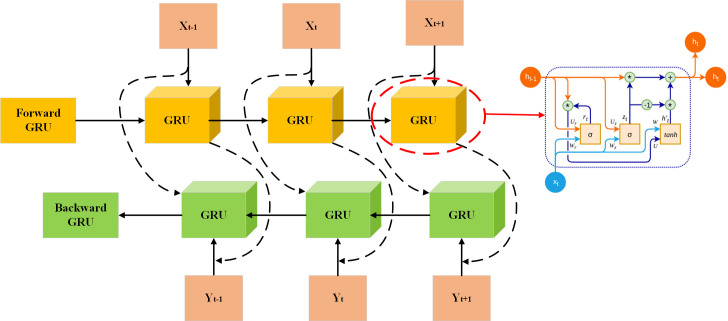
Bidirectional gated recurrent unit architecture.

The BiGRU is a specialized type of recurrent neural network (RNN) designed to work on sequence data in directions of forward and backward, as depicted in Fig 4. By integrating both forward and backward GRUs, this architecture enables the model to account for both past and future information at each time step. By processing data in both directions, the model is much better able to capture and model intricate patterns within sequential data. As such, BiGRU is particularly well-suited for tasks that require a deep understanding of sequential context, like time series forecasting and natural language processing.

In the BiGRU framework, the hidden state at each time step, is derived from the hidden states of both the forward and backward GRUs. The forward GRU processes the input sequence from the start to the end (from *t* = 1 to *t* = *T*), producing the forward hidden state sequence. In contrast, the backward GRU processes the sequence in reverse order (from *t* = *T* to *t* = 1), resulting in the backward hidden state sequence. Then, at every interval, the final hidden state $h_{t}$, is typically obtained by concatenating or combining the forward and backward hidden states, thus leveraging information from both directions.

The mathematical representation of the forward Gated Recurrent Unit (GRU) is expressed as follows:


$$z_{t}=\sigma (W_{z}\cdot \left[h_{t-1},x_{t}\right]+f_{z})$$ (14)

$$r_{t}=\sigma (W_{r}\cdot \left[h_{t-1},x_{t}\right]+f_{r})$$ (15)

$$h_{t}^{\prime }=tanh\left(W_{h}\cdot \left[{r_{t}⨀h}_{t-1},x_{t}\right]+f_{h}\right)$$ (16)

$$h_{t}=\left(1-c_{t}\right)⨀h_{t-1}+c_{t}⨀h_{t-1}^{\prime }$$ (17)

And the mathematical representation of the backward Gated Recurrent Unit (GRU) is expressed as follows:


$$\overset{\leftarrow }{z_{t}}=\sigma (W_{\overset{\leftarrow }{z}}\cdot \left[{\overset{\leftarrow }{h}}_{t-1},x_{t}\right]+f_{\overset{\leftarrow }{z}})$$ (18)

$$\overset{\leftarrow }{r_{t}}=\sigma (W_{\overset{\leftarrow }{r}}\cdot \left[{\overset{\leftarrow }{h}}_{t-1},x_{t}\right]+f_{\overset{\leftarrow }{r}})$$ (19)

$$\overset{\leftarrow }{h_{t}^{\prime }}=tanh(W_{\overset{\leftarrow }{h}}\cdot \left[\overset{\leftarrow }{r_{t}}⨀{\overset{\leftarrow }{h}}_{t-1},x_{t}\right]+f_{\overset{\leftarrow }{h}})$$ (20)

$$\overset{\leftarrow }{h_{t}}=\left(1-\overset{\leftarrow }{c_{t}}\right)⨀{\overset{\leftarrow }{h}}_{t-1}+\overset{\leftarrow }{c_{t}}⨀{\overset{\leftarrow }{h}}_{t-1}$$ (21)

In the above formula, $W_{z}$, $W_{r}$ and $W_{h}$ refer to the weight matrices for the forward GRU, while ${\overset{\leftarrow }{W}}_{z}$, ${\overset{\leftarrow }{W}}_{r}$ and ${\overset{\leftarrow }{W}}_{h}$ refer to the weight matrices for the backward GRU. $f_{z}$, $f_{r}$, $f_{h}$, $f_{\overset{\leftarrow }{z}}$, $f_{\overset{\leftarrow }{r}}$, $f_{\overset{\leftarrow }{h}}$ denote the bias terms. The sigmoid function, denoted by σ, is utilized as the activation function, and the operator ⨀ indicates element-wise multiplication.

### 2.6. Attention mechanism(AM)

The attention mechanism (AM) is a computational framework that aims to replicate human visual attention patterns. Its core function is to selectively concentrate on the most relevant portions of the data being processed, therefore improving both processing efficiency and the accuracy of the model. This is accomplished by incorporating an attention vector, which assigns dynamic weights to different parts of the input data derived from its current characteristics. Being calculated in real-time, these attention weights can allow the model to prioritize the most significant data segments while minimizing the influence of less relevant information. The weight allocation formula for AM is in the following manner:


$${score}_{t,s}=\text{tanh}(w_{t}h_{t}+w_{s}h_{s})$$ (22)

$$a_{i}=softmax\left({score}_{i,s}\right)=\frac{\text{exp}({score}_{i,s})}{\sum_{j=1}^{\tau }\text{exp}({score}_{j,s})}$$ (23)

$$a=\sum_{i=1}^{\tau }a_{i}h_{i}$$ (24)

Where ${score}_{t,s}$ represents the attention probability distribution function, $w_{t}$ and $w_{s}$ are the attention weight matrices, while $h_{t}$ and $h_{s}$ correspond to the hidden layer vectors. The variable $a_{i}$ signifies the attention score and a is the output value from the attention layer.

### 2.7. BKA-VMD-1DCNN-BiTCN-BiGRU-attention model

**Fig 5:**
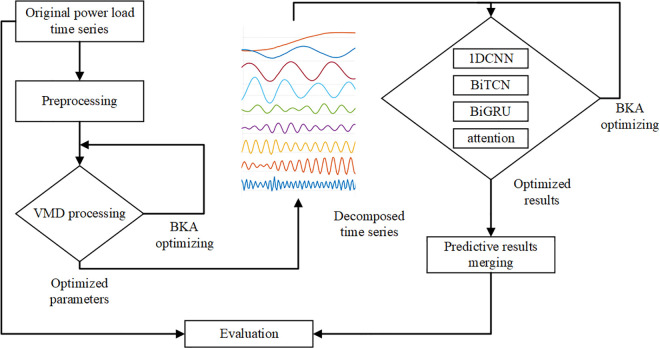
Algorithmic flow of BKA-VMD-1DCNN-BiTCN-BiGRU-attention model.

To address the complexities of industrial power load forecasting, we propose a hybrid deep learning model which integrates 1D Convolutional Neural Networks (1DCNN), Bidirectional Temporal Convolutional Networks (BiTCN), Bidirectional Gated Recurrent Units (BiGRU), and an attention mechanism. This combination is strategically chosen to leverage the unique strengths of each component in processing time series data. The 1DCNN excels at extracting local temporal features, crucial for capturing short-term patterns in power load data. Building on these features, the BiTCN employs dilated convolutions to model long-range dependencies, effectively handling seasonal variations and extended sequences. The BiGRU further refines the model by learning bidirectional sequential patterns, ensuring a comprehensive understanding of both past and future contexts. Finally, the attention mechanism dynamically focuses on the most relevant features, enhancing prediction accuracy by weighting critical time steps. Mechanistically, the data flows sequentially: preprocessed time series data, decomposed using Variational Mode Decomposition (VMD) optimized by the Black-Winged Kite Algorithm (BKA), is first processed by the 1DCNN for local feature extraction. The resulting feature maps are then passed to the BiTCN to capture long-range temporal dependencies, followed by the BiGRU to model sequential dependencies bidirectionally. The attention mechanism then weights the BiGRU outputs, emphasizing the most informative features for final power load predictions. This structured integration, as illustrated in Fig 5, ensures that each component contributes uniquely to producing accurate and reliable forecasts.

The start of the proposed model is receiving the original power load time series data, which undergoes preprocessing, including Variational Mode Decomposition (VMD). This decomposition process splits the time series into its intrinsic modes, enabling the model to capture detailed information across various frequencies. Subsequently, the data is passed to the BKA stage, where key parameters are optimized to enhance predictive performance. The decomposed time series data is then processed through the 1D-CNN, BiTCN, and BiGRU layers, each of which captures forward and backward temporal dependencies within the data. An attention mechanism is then applied to emphasize the most significant parts of the data, further improving the model’s ability to make precise predictions. The output from the attention layer is later aggregated to generate the final predictions. Furthermore, in order to maximize the model’s efficiency, the BKA is applied once more to refine the parameters based on the evaluation results, thereby ensuring the model’s robustness and accuracy across various power load scenarios.

## 3. Results and discussion about proposed model

### 3.1. Details of power load dataset

To assess the accuracy and generalizability of the proposed model, we have prepared three different power load datasets in industry of vehicle or mechanical manufacturing. The first power load dataset is collected from the vehicle transmission manufacturing workshop at Liuzhou Saike Technology Company. The second power load dataset is collected from the product testing center in the same company. And the third power load dataset is collected from the mechanical product assembly workshop at Guangxi Liugong Machinery Co., Ltd. All of the workshops are equipped with smart meters, all of which are managed by an intelligent energy management system. This system ensures the continuous accumulation of power load data generated throughout the manufacturing process on a daily basis. The data spans from 20 September 2022–15 December 2023, comprising 452 sampling timestamps. To construct the training and validation datasets, all of the original datasets is partitioned into two subsets, with 70% allocated for training and 30% for validation.

**Fig 6:**
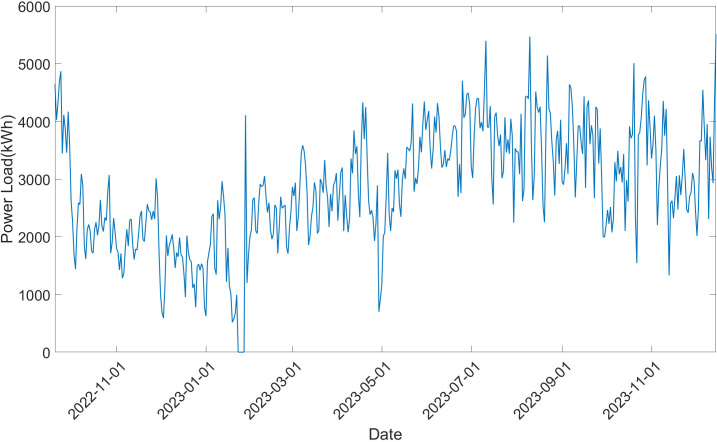
Original power load time series diagram of the first dataset.

The details of first dataset are depicted as Fig 6. Due to potential interruptions in the factory’s manufacturing schedule during the Spring Festival, it was observed that the power load could drop to zero during this period. Consequently, the data corresponding to this week was excluded from the analysis. The remaining dataset consists of 447 sampling timestamps. We can see that there exist many components in power load of the factory. Therefore, it is necessary to perform the decomposition to the original power load time series to make the prediction more accuracy.

### 3.2. Selection of evaluation indicators

For the purpose of comprehensively evaluating the accuracy of power load predictions generated by the proposed models, this study adopts two standard performance metrics: the Mean Absolute Error (MAE) and the Root Mean Square Error (RMSE). These metrics serve to quantify the differences between the predicted outcomes and the actual values, thus providing an objective means for examining the model’s performance. The MAE measures the average of the absolute deviations between the predicted and observed values, as described by the following formula:


$$MAE=\frac{1}{N}\sum_{i=1}^{N}\left|y_{i}-\hat{y_{i}}\right|$$ (25)

In this formula, *N* represents the total number of samples, $\hat{y_{i}}$ refers to the predicted value, and $y_{i}$ signifies the actual value. A reduction in MAE indicates a corresponding improvement in the model’s prediction accuracy.

RMSE is the average of the squared discrepancies between the actual and predicted values. Its formula is as follows:


$$RMSE=\sqrt{\frac{1}{N}\sum_{i=1}^{N}{\left(y_{i}-\hat{y_{i}}\right)}^{2}}$$ (26)

Like MAE, lower RMSE values signify better model performance; however, it is particularly sensitive to large errors, which makes it highly relevant when evaluating datasets with considerable variability. By conducting an in-depth comparison of both MAE and RMSE, we can acquire a deeper comprehension of the predictive capabilities of the proposed model, thereby providing a solid foundation for future model refinement and optimization.

### 3.3. VMD parameters optimization via BKA

The selection of appropriate parameters for Variational Mode Decomposition (VMD) largely depends on practical experience, with the α (modes) and K (moderate bandwidth) parameters playing a critical role in determining the effectiveness of the decomposition process. Specifically, the value of K is closely connected with the efficiency of the modal decomposition. A significantly elevated K value may result in the loss of key modal characteristics, while a value that is too low could lead to frequency aliasing. On the other hand, the penalty factor α primarily regulates the bandwidth of the modes derived from the decomposition. Increasing α helps minimize the bandwidth but may lead to an inaccurate central frequency, while reducing α could introduce excessive noise, affecting the quality of the estimated modes.

As a result, the BKA can be adopted to optimize the parameters [*α*,] in the VMD process. We choose mean absolute percentage error(MAPE) as the fitness function of BKA, the formula can be shown as:


$$MAPE=\frac{100\%}{N}\sum_{i=1}^{n}\left|\frac{\hat{y_{i}}-y_{i}}{y_{i}}\right|$$ (27)

**Fig 7:**
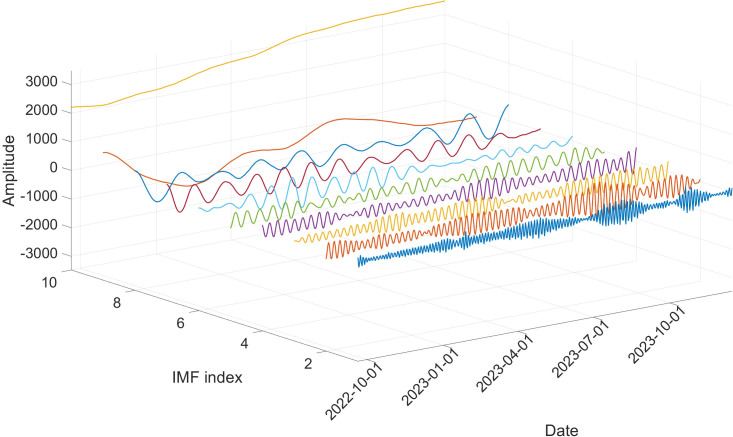
Original power load time series decomposed result.

In the BKA, the assessment of MAPE can be seen as the target function. This marks the point at which the optimal position of the leading black-winged kite is identified, indicating the algorithm has successfully converged to the most effective solution. Following this, the optimized values for α and K are employed as the initial parameters for VMD, which are used to decompose the data for power load time series. Fig 7 illustrates the decomposition results obtained for the data from original power load time series.

### 3.4. Comparison of different deep learning model

After decomposing the original power load time series into Intrinsic Mode Functions (IMFs), all resulting IMFs are fed as inputs into the core module of the deep learning model to generate predictive results. These individual predictions are then merged to reconstruct the predicted power load time series. To evaluate the performance of the proposed model and compare with alternative hybrid models, an extensive ablation study was conducted. The algorithms considered in this study include 1DCNN-BiTCN-BiGRU, 1DCNN-TCN-GRU, 1DCNN-BiGRU, 1DCNN-BiTCN, and BiTCN-BiGRU, in addition to the model proposed in this research. The training dataset was employed to train both the proposed model and the comparison models, while the validation dataset was employed to assess whether our model outperforms the other hybrid deep learning models in the task of power load prediction.

Table 2 presents the evaluation metrics, specifically Mean Absolute Error (MAE) and Root Mean Square Error (RMSE), for each algorithm across the three datasets. The proposed 1DCNN-BiTCN-BiGRU-AM model consistently achieves the lowest MAE and RMSE values across all datasets, demonstrating its superior predictive accuracy and stability across diverse industrial scenarios.

**Table 2:** Comparison results of different deep learning models.

Dataset	Models	MAE	RMSE
Dataset 1	BiTCN-BiGRU	590.380	742.929
	1DCNN-BiTCN	571.874	723.404
	1DCNN-BiGRU	558.691	709.801
	1DCNN-TCN-GRU	595.665	740.870
	1DCNN-BiTCN-BiGRU	549.271	688.054
	1DCNN-BiTCN-BiGRU-AM	**537.222**	**677.989**
Dataset 2	BiTCN-BiGRU	235.032	301.076
	1DCNN-BiTCN	227.344	296.484
	1DCNN-BiGRU	224.452	301.713
	1DCNN-TCN-GRU	251.644	330.405
	1DCNN-BiTCN-BiGRU	215.792	280.739
	1DCNN-BiTCN-BiGRU-AM	**205.440**	**272.423**
Dataset 3	BiTCN-BiGRU	1345.599	1588.452
	1DCNN-BiTCN	1199.265	1622.190
	1DCNN-BiGRU	1260.171	1716.691
	1DCNN-TCN-GRU	1443.304	1635.010
	1DCNN-BiTCN-BiGRU	1242.837	1648.461
	1DCNN-BiTCN-BiGRU-AM	**1110.976**	**1511.251**

In particular, when compared to hybrid models that do not incorporate the 1DCNN structure, such as the BiTCN-BiGRU model, the proposed model shows substantial improvements. For the first dataset, the reductions in MAE and RMSE are approximately 9.00% and 8.74%, respectively. For the second dataset, the improvements are 12.59% in MAE and 9.52% in RMSE, and for the third dataset, 17.43% in MAE and 4.86% in RMSE. These results highlight the effectiveness of the 1DCNN component in capturing local temporal patterns and enhancing feature extraction from sequential data.

Moreover, the benefits of employing bidirectional networks are evident when comparing the 1DCNN-BiTCN-BiGRU model with its unidirectional counterpart, 1DCNN-TCN-GRU. For the first dataset, the bidirectional model reduces MAE by 7.79% and RMSE by 7.13%. Similarly, for the second dataset, the improvements are 14.25% in MAE and 15.03% in RMSE. For the third dataset, while the bidirectional model shows a slight increase in RMSE (0.82%), the proposed model with the attention mechanism achieves a lower RMSE (1511.251 compared to 1635.010), indicating that the attention mechanism mitigates this limitation.

The integration of the attention mechanism in the proposed model further enhances its predictive capabilities. Compared to the 1DCNN-BiTCN-BiGRU model, the addition of attention leads to additional reductions in MAE and RMSE: 2.19% and 1.46% for the first dataset, 4.80% and 2.96% for the second, and 10.61% and 8.32% for the third dataset, respectively. This underscores the attention mechanism’s ability to focus on the most relevant features, thereby improving the model’s accuracy and robustness across varied industrial contexts.

In conclusion, the proposed 1DCNN-BiTCN-BiGRU-AM model demonstrates superior performance over other advanced hybrid models across multiple industrial power load datasets. The synergistic combination of 1DCNN for local feature extraction, bidirectional temporal convolutional and recurrent networks for capturing comprehensive temporal dependencies, and the attention mechanism for feature prioritization contributes to its enhanced predictive accuracy, generalizability, and stability. These results affirm the model’s robustness in handling diverse industrial scenarios, addressing the need for reliable power load forecasting in complex manufacturing environments.

**Fig 8:**
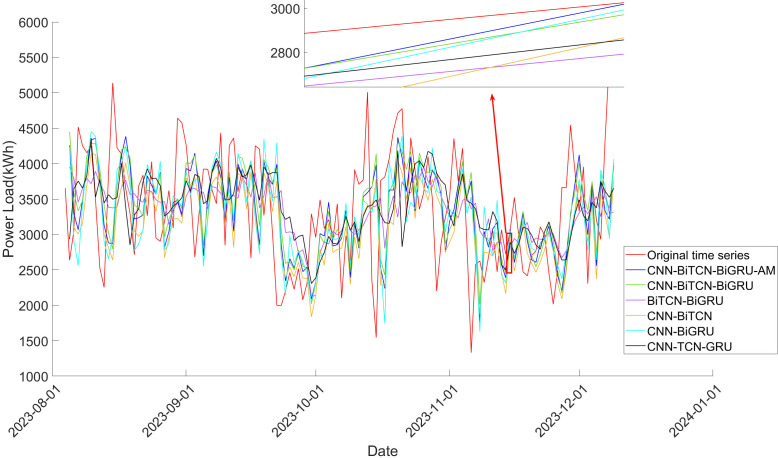
Comparison of prediction result of models on decomposed power load time series in Dataset 1.

**Fig 9:**
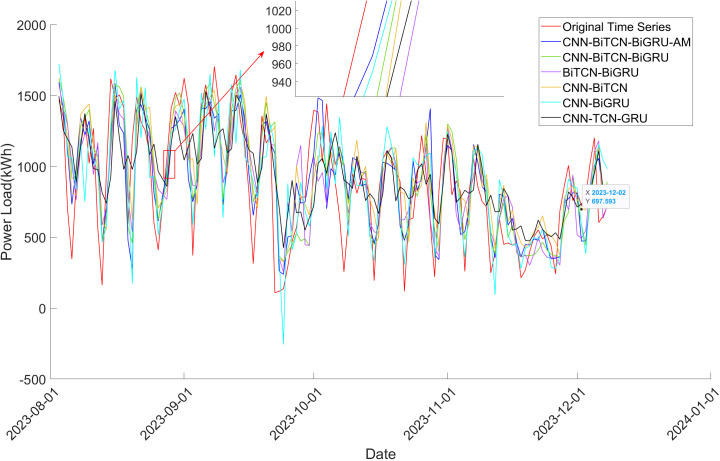
Comparison of prediction result of models on decomposed power load time series in Dataset 2.

**Fig 10:**
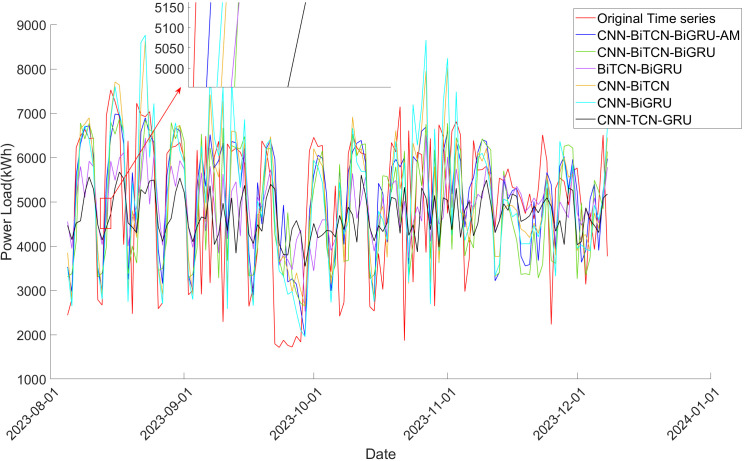
Comparison of prediction result of models on decomposed power load time series in Dataset 3.

Figs 8–10 provides a further comparative analysis of the prediction outcomes across the algorithms. It is clear from looking at the graphs that the 1DCNN-BiTCN-BiGRU-AM model offers the best fit to the original time series, exhibiting fewer fluctuations. This observation underscores the importance of the bidirectional feature extraction mechanism, both local and long-term, which substantially improves the accuracy and stability of power load prediction.

### 3.5. Comparison of different decomposition methods

To assess the impact of various decomposition methods on the performance of our proposed power load prediction model, we conducted a comparative analysis using four approaches: the baseline model without decomposition (CNN-BiTCN-BiGRU-Attention) and the model integrated with Empirical Mode Decomposition (EMD), Empirical Wavelet Transform (EWT), and Variational Mode Decomposition (VMD). The evaluation was performed across three distinct datasets, utilizing Mean Absolute Error (MAE) and Root Mean Square Error (RMSE) as the primary metrics to gauge prediction accuracy.

The results, as presented in Table 3, demonstrate that incorporating decomposition methods significantly enhances prediction accuracy compared to the baseline model. The VMD-based model consistently achieved the lowest MAE and RMSE across all datasets. For Dataset1, the VMD model reduced the MAE by approximately 43.2% (from 594.786 to 338.147) and the RMSE by 42.3% (from 753.465 to 434.769) relative to the baseline. In Dataset2, the improvements were 49.3% in MAE (from 207.190 to 105.137) and 49.6% in RMSE (from 276.869 to 139.528). For Dataset3, the VMD model exhibited reductions of 69.3% in MAE (from 1182.207 to 363.561) and 70.3% in RMSE (from 1563.628 to 464.740). While EMD and EWT also improved performance, with EWT achieving a notable 53.4% MAE reduction in Dataset3, their enhancements were less pronounced compared to VMD. These findings underscore the superior capability of the VMD decomposition method, in effectively capturing the complex patterns in power load time series data, thereby significantly enhancing the predictive performance of the model.

**Table 3:** Comparison results of different decomposition methods.

Dataset	Models	MAE	RMSE
Dataset1	CNN-BiTCN-BiGRU-Attention	594.786	753.465
	EMD-CNN-BiTCN-BiGRU-Attention	493.366	630.619
	EWT-CNN-BiTCN-BiGRU-Attention	508.519	652.923
	VMD-CNN-BiTCN-BiGRU-Attention	**338.147**	**434.769**
Dataset2	CNN-BiTCN-BiGRU-Attention	207.190	276.869
	EMD-CNN-BiTCN-BiGRU-Attention	193.120	250.688
	EWT-CNN-BiTCN-BiGRU-Attention	192.441	251.309
	VMD-CNN-BiTCN-BiGRU-Attention	**105.137**	**139.528**
Dataset3	CNN-BiTCN-BiGRU-Attention	1182.207	1563.628
	EMD-CNN-BiTCN-BiGRU-Attention	1080.416	1397.477
	EWT-CNN-BiTCN-BiGRU-Attention	550.766	700.848
	VMD-CNN-BiTCN-BiGRU-Attention	**363.561**	**464.740**

### 3.6. Comparison of different optimization methods

To further address the need for a comprehensive comparison with advanced hybrid models, we evaluate the impact of various optimization algorithms on the base model (VMD-1DCNN-BiTCN-BiGRU-AM) identified in section 3.4 as the best-performing architecture. The optimization algorithms tested include the Black-Winged Kite Algorithm (BKA), Whale Optimization Algorithm (WOA), Ant Lion Optimizer (ALO), Grey Wolf Optimizer (GWO), Dragonfly Algorithm (DA), and Moth-Flame Optimization (MFO). These algorithms fine-tune both the Variational Mode Decomposition (VMD) parameters and the deep learning model’s hyperparameters, such as learning rate, neuron numbers, and attention settings. The results, presented in Table 3, compare the Mean Absolute Error (MAE) and Root Mean Square Error (RMSE) across three industrial power load datasets.

Table 4 demonstrates that parameter optimization significantly enhances the predictive accuracy of the base model. The BKA-optimized model consistently achieves the lowest MAE and RMSE across all datasets. For Dataset 1, the base model yields an MAE of 354.377 and an RMSE of 444.743, while BKA optimization reduces these to 119.451 and 154.615, respectively, corresponding to improvements of 66.3% in MAE and 65.2% in RMSE. For Dataset 2, BKA reduces MAE by 29.6% (from 158.158 to 111.318) and RMSE by 27.2% (from 197.110 to 143.486). For Dataset 3, the improvements are even more pronounced, with MAE decreasing by 67.4% (from 650.759 to 212.370) and RMSE by 65.8% (from 849.617 to 290.175).

**Table 4:** Comparison results of different optimization methods.

Dataset	Models	MAE	RMSE
Dataset 1	Base model	354.377	444.743
	BKA+Base model	**119.451**	**154.615**
	WOA+Base model	271.334	335.437
	ALO+Base model	309.914	393.389
	GWO+Base model	153.462	191.913
	DA+Base model	199.276	255.098
	MFO+Base model	344.400	436.857
Dataset 2	Base model	158.158	197.110
	BKA+Base model	**111.318**	**143.486**
	WOA+Base model	144.933	182.837
	ALO+Base model	125.772	167.941
	GWO+Base model	124.580	161.862
	DA+Base model	144.018	182.525
	MFO+Base model	126.670	161.710
Dataset3	Base model	650.759	849.617
	BKA+Base model	**212.370**	**290.175**
	WOA+Base model	360.128	466.042
	ALO+Base model	671.474	904.763
	GWO+Base model	670.774	910.355
	DA+Base model	663.557	901.116
	MFO+Base model	500.416	604.759

Comparing BKA with other optimization algorithms, BKA consistently outperforms its counterparts. For Dataset 1, BKA’s MAE of 119.451 is 22.2% lower than GWO’s 153.462, the next best optimizer, and significantly better than WOA (271.334), ALO (309.914), DA (199.276), and MFO (344.400). Similar trends are observed in Dataset 2, where BKA’s MAE is 10.7% lower than GWO’s 124.580, and in Dataset 3, where BKA’s MAE is 41.0% lower than WOA’s 360.128. Notably, for Dataset 3, algorithms like ALO, GWO, and DA yield higher errors than the base model, suggesting that suboptimal parameter tuning can degrade performance. BKA’s superior performance likely stems from its effective balance of exploration and exploitation, enabling it to identify optimal parameter combinations for both VMD and the deep learning architecture. The optimized parameters, detailed in Table 5, include a learning rate of 0.004, 8 neurons, a dropout factor of 0.1, 1 attention head, 2 attention keys, 4 VMD modes, and a VMD moderate bandwidth of 101.

**Table 5:** Optimized parameters.

Parameters	Optimized Value
Learning rate	0.004
Neuron numbers	8
Dropout factor	0.1
Attention heads	1
Attention keys	2
VMD modes	4
VMD moderate bandwidth	101

In conclusion, the integration of BKA with the VMD-1DCNN-BiTCN-BiGRU-AM model significantly enhances its predictive accuracy, establishing it as a superior choice for power load forecasting compared to other optimization algorithms. This analysis underscores the importance of effective parameter optimization in achieving robust performance across diverse industrial scenarios, further highlighting the innovativeness and superiority of the proposed model.

And we also analyze the computational complexity of our proposed model, focusing on inference time for practical applications like industrial power load forecasting. The model’s inference complexity is dominated by the attention mechanism, with a time complexity of O(T^2^), where T is the sequence length. Since the Variational Mode Decomposition algorithm decompose the original sequence into K modes, the computational complexity of combined VMD and base model is O(KT^2^). And according to Wang et al. [33], the computational complexity of Blackwinged Kite Algorithm(BKA) is O(K·(T+T·D+1)), where K is the number of decomposed modes, and T is the maximum number of iterations and D is the specific problem’s dimension. Therefore, the combined computational complexity of our proposed model is $O(K^{2}T^{4}\cdot (T+T\cdot D+1))$.

## 4. Conclusion and discussion

Power load prediction is crucial in various scenarios, such as manufacturing within a factory, where it can significantly contribute to more efficient production planning, minimizing energy wastage. However, predicting such time series data in real-world applications can be challenging due to the complex components inherent in the original time series. This research suggested a novel power load prediction model that combines an optimized data decomposition method with a hybrid deep learning framework, making the following key contributions:

1. We introduce a VMD-based method, optimized using the BKA, to decompose the original time series into distinct IMFs. To enhance the model’s performance, the BKA is further employed to optimize the parameters of the hybrid deep learning model. Compared to models without parameter optimization or data decomposition, our proposed model achieves superior prediction performance. This demonstrates that the BKA is an effective approach for enhancing model performance in time series prediction tasks.
2. Distinct from traditional prediction models, our proposed approach integrates both forward and reverse temporal features to enhance forecasting accuracy. This is achieved through a multi-layered architecture: a One-Dimensional Convolutional Neural Network (1DCNN) first captures local temporal patterns and key features in the sequential data. Subsequently, a Bidirectional Temporal Convolutional Network (BiTCN) extracts bidirectional temporal features, leveraging information from both past and future contexts. A Bidirectional Gated Recurrent Unit (BiGRU) then models long-term dependencies in both directions. An attention mechanism further refines the model’s focus by weighting the most informative parts of the input data. The effectiveness of this integrated approach is confirmed through ablation studies. In comparative evaluations with state-of-the-art models, our model consistently achieves lower Mean Absolute Error (MAE) and Root Mean Square Error (RMSE) across all datasets. For instance, on Dataset 1, it outperforms the BiTCN-BiGRU model by 9.00% in MAE and 8.74% in RMSE, the 1DCNN-BiTCN model by 6.06% and 6.28%, the 1DCNN-BiGRU model by 3.84% and 4.48%, the 1DCNN-TCN-GRU model by 9.81% and 8.49%, and the 1DCNN-BiTCN-BiGRU model by 2.19% and 1.46%, respectively. These improvements highlight the model’s enhanced robustness and reliability in capturing underlying data trends, thereby improving prediction accuracy and stability.

However, there also exist some limitations on our research, and future work should focus on coping with such limitations. First the hybrid deep learning architecture, incorporating 1D Convolutional Neural Networks (1DCNN), Bidirectional Temporal Convolutional Networks (BiTCN), Bidirectional Gated Recurrent Units (BiGRU), and attention mechanisms, is computationally intensive. This complexity may pose challenges for real-time deployment or application in environments with limited computational resources. Future work could focus on optimizing the model to reduce its computational footprint while maintaining accuracy. Second, the datasets consist of 447 daily sampling timestamps, which, although sufficient for capturing seasonal patterns, may not be large enough to fully leverage the capabilities of deep learning models, potentially leading to overfitting. In future work we will try to expand the dataset or employ data augmentation techniques to help mitigate this issue. Finally, the study did not account for external factors such as weather conditions, holidays, or production schedules, which can significantly impact power load. We will incorporate these variables could enhance the model’s predictive accuracy and robustness.

## Supporting information

S1 File Data. (ZIP)
